# Validity and responsiveness of four measures of occupational sitting and standing

**DOI:** 10.1186/s12966-015-0306-1

**Published:** 2015-11-25

**Authors:** Femke van Nassau, Josephine Y. Chau, Jeroen Lakerveld, Adrian E. Bauman, Hidde P. van der Ploeg

**Affiliations:** Department of Public and Occupational Health, EMGO Institute for Health and Care Research, VU University Medical Center, Van der Boechorststraat 7, 1081BT Amsterdam, The Netherlands; Sydney School of Public Health, University of Sydney, L6 Charles Perkins Centre, University of Sydney, Sydney, NSW 2006 Australia; Department of Epidemiology and Biostatistics, EMGO Institute for Health and Care Research, VU University Medical Center, Van der Boechorststraat 7, 1081 BT Amsterdam, The Netherlands

**Keywords:** Sitting, Standing, Measurement, Occupational health, Sedentary behavior

## Abstract

**Background:**

Evidence on the detrimental health effects of prolonged sedentary behavior is accumulating. Interventions need to have a specific focus on sedentary behavior in order to generate clinically meaningful decreases in sedentary time. When evaluating such intervention, the question whether a participant improved or deteriorated their behavior is fundamental and instruments that are able to detect those changes are essential. Therefore, the aim of this study was to determine the criterion validity against activPAL and responsiveness to change of two activity monitors (ActiGraph and activPAL) and two questionnaires for the assessment of occupational sitting and standing time.

**Methods:**

42 participants took part in the Stand@Work intervention trial. Six (T0) and two (T1) weeks before they received a sit-stand workstation and three weeks thereafter (T2), participants wore an ActiGraph and an activPAL activity monitor, and completed the Occupational Sitting and Physical Activity Questionnaire (OSPAQ) and the Workforce Sitting Questionnaire (WSQ). The activPAL was used as the criterion validity measure.

**Results:**

The ActiGraph showed strong validity for occupational sedentary time at T0 and T1 (Spearman rho = 0.77 and 0.69), but its validity dropped substantially after introduction of the sit-stand workstation (rho = 0.19). Correlations between occupational light-intensity activity assessed by the ActiGraph and occupational standing time assessed by the activPAL varied between 0.25–0.63. The occupational sitting validity correlation of the OSPAQ and WSQ varied from 0.35-0.48 and 0.25-0.30, respectively, and between 0.16–0.68 for the OSPAQ for occupational standing time. The intervention-induced changes in occupational sitting and standing time were well detected by the activPAL, OSPAQ and WSQ (sitting only), but not by the ActiGraph, which had the lowest responsiveness to change.

**Conclusions:**

This study suggests that studies aimed at determining differences in occupational sitting and standing time should use activPAL-type inclinometers as a preferred type of objective measure. Simple questionnaires showed sufficient validity and are usable in addition to an objective measure or alone when objective monitoring is not possible. The hip-worn ActiGraph was unable to distinguish between occupational sitting and standing time, when using uniaxial data and traditional cut-points for sedentary time and light-intensity activity.

**Trial registration:**

The study was registered with the Australian New Zealand Clinical Trials Registry (No. ACTRN 12612000072819).

## Background

Evidence on the detrimental health effects of prolonged sedentary behavior is accumulating [1–4]. Large epidemiological studies suggest that high volumes of sitting time are associated with all-cause, cardiovascular and possibly cancer-mortality [5–8].

Many adults in developed countries spend an extensive amount of their work time sitting, and hence the workplace is regarded as a suitable setting to interrupt those prolonged sitting periods [9–11]. Several interventions have been implemented in the occupational setting, but were primarily physical-activity focused [12]. Evidence shows that interventions need to have a specific focus on sedentary behavior in order to generate decreases in sedentary time [13]. As such, sit-stand workstations specifically aim to reduce sitting time by permitting users to alternate between sitting with standing [14–16]. Neuhaus et al. concluded that the introduction of sit-stand workstations could reduce occupational sedentary time without compromising work performance [17].

In order to evaluate the effectiveness of workplace interventions, both subjective and objective measurement instruments are being used to measure (changes in) sitting and standing behavior [18]. Examples of subjective measures include 3-day activity diaries [19], previous-day recall interviews [20] or the interviewer-administered Past-day Adults’ Sedentary Time (PAST) [21]. Furthermore, single or multiple item self-administered questionnaires such as the International Physical Activity Questionnaire (IPAQ) [22], Marshall questionnaire [23], Workforce Sitting Questionnaire (WSQ) [24], and the Occupational Sitting and Physical Activity Questionnaire (OSPAQ) [25] have also been used. These self-report methods are relatively cheap, easy to use, and can be administered on a large scale, but are susceptible to social desirability and recall bias.

Objective measures using accelerometers and inclinometers can assess sitting and standing time objectively. Commonly used devices are the ActiGraph accelerometer (ActiGraph, LLC, Fort Walton Beach, FL ), often worn on the hip or wrist, and the thigh-worn activPAL inclinometer (PAL Technologies Ltd., Glasgow, UK). The activPAL is widely considered the most accurate method for assessing sitting posture and has shown high agreement compared with direct observation [26].

When evaluating intervention effects, the question whether a participant improved or deteriorated their behavior is fundamental and instruments that are able to detect those changes are essential. Only a few studies have examined responsiveness to change in sedentary time in adults [21, 27–29], of which some reported the activPAL to be the most sensitive measure for detecting changes [27, 28]. Others reported lack of responsiveness of the activPAL [21] or no difference between the activPAL and ActiGraph [29].

The responsiveness of objectively assessed and self-reported changes in the sit-stand transition following the introduction of a sit-standing workstation has not yet been examined. In order to determine the responsiveness of both objective and subjective methods, we used data from the Australian Stand@Work trial that evaluated the effectiveness of sit-stand workstations on office workers’ sitting time [30, 31]. That study showed that the introduction of sit-stand workstations reduced office workers’ sitting time by 73 min/workday (95 % CI = −106; −39) and increased standing time by 65 min/workday (95 % CI = 47; 83) as measured by activPAL [31]. Since the trial resulted in significant intervention effects, it was deemed suitable to determine the responsiveness to change of four different assessment methods.

The aim of our study was twofold: 1) to determine the criterion validity of the ActiGraph accelerometer, OSPAQ and WSQ to assess occupational sitting and standing time compared to the activPAL, and 2) to determine the responsiveness of the activPAL, ActiGraph, OSPAQ and WSQ to changes in occupational sitting and standing time following the successful introduction of a sit-stand workstation.

## Methods

Data used for this study were drawn from the Stand@Work trial of which details can also be found elsewhere [30, 31]. The study was approved by the University of Sydney Human Research Ethics Committee (No. 08-2011/14067) and all participants provided written informed consent. The study was registered with the Australian New Zealand Clinical Trials Registry (No. ACTRN 12612000072819).

### Participants

Participants were staff from a non-government health agency in Sydney, Australia, aged over 18 years, employed at least three days per week, and who had sufficient English language proficiency to complete the study materials. The project was advertised to staff as part of their workplace wellness program via internal mail, staff meetings and information fliers in the office. Staff members who were interested could join the study by returning an expression of interest form.

### Design

The trial used a randomized controlled crossover design with a waitlist control group and rolling recruitment. Eligible staff members who returned an expression of interest form were randomly drawn from a ballot and assigned to an intervention or waiting list control group condition in groups of four to five people. After four weeks, the first waiting list control group received the intervention and the second waitlist group served as their control group. This process was repeated until nine groups had received the intervention. Data collection ran from September 2011 to July 2012. More details about the study design are described elsewhere [30, 31].

### Intervention

Those in the intervention group were provided with a sit-stand workstation (Ergotron Workfit S) to use at work for four weeks. The sit-stand workstation allowed office workers to vary their posture throughout the workday between sitting and standing. Prior to receiving the sit-stand workstation, participants received brief instructions on its use.

## Measures

**Fig. 1:**
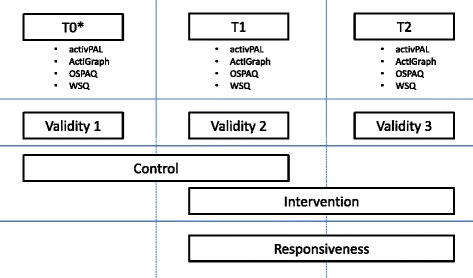
Design of the study. *No data for the first intervention group, which only participated in T1 and T2, because they could not serve as a time synchronized control condition

Both objective and self-report measures of occupational sitting and standing time were collected. Assessments took place at three time points scheduled four weeks apart. Assessment 1 (T0) was six weeks pre-intervention, assessment 2 (T1) at two weeks pre-intervention and assessment 3 (T2) during the third week of the intervention (Fig. 1).

Changes between T0 and T1 were treated as the control condition, changes between T1 and T2 as the intervention condition. The exception was the first wave of 4 participants that were allocated to the intervention group, which only participated in T1 and T2, because they could not serve as a time synchronized control condition.

During all three assessments, participants wore two activity monitors (activPAL and ActiGraph). The two devices were worn during work hours for the working week. Participants kept a monitoring logbook to note the times they wore the monitors each day, the days they worked, and the times they started and finished work on each of those days.

The *activPAL activity monitor* (model activPAL3; PAL Technologies Ltd., Glasgow, UK) is a small (5 × 3.5 × 0.7 cm) monitor that weighs 20 g and was worn on the front of the thigh during working hours. The activPAL measures time spent sitting, standing and stepping. ActivPAL data were first processed using proprietary software (activPAL v6.1.2.17, PAL Technologies Ltd., Glasgow, UK) and custom software (HSC analysis software v2.19, Philippa Dall and Malcolm Granat, Glasgow Caledonian University), which allowed for the isolation of participants’ work time data based on their work start and finish times reported in their monitoring log.

The *ActiGraph GT1M and GT3X activity monitor* (ActiGraph, LLC, Fort Walton Beach, FL) are commonly used small (51 × 41 × 15 mm), lightweight (27 grams) uniaxial accelerometers. The ActiGraph was worn on the right hip during waking hours in the same week the activPAL was worn. The ActiGraph measures activity counts, which can be converted into time spent in sedentary, light, moderate and vigorous intensity activities. Non-wear time was defined as 60 minutes of consecutive zeroes, allowing for two interruptions of <100 counts per minute. Time spent in sedentary (<100 counts/min), light (100–2019 counts/min), moderate (2020–5998 counts/min), and vigorous intensity activity (>5999 counts/min) were calculated with established cut points for adults [32]. Activity during work hours was determined by temporally linking the activPAL and ActiGraph data to the work times recorded in the monitoring log book.

Participants also completed two questionnaires of which the recall period matched the period the activity monitors were worn. The *Occupational Sitting and Physical Activity Questionnaire* (OSPAQ) asks participants to indicate the proportion of their working time on a typical workday in the last 7 days that they spent sitting, standing, walking, and doing heavy labor or physically demanding tasks during work time [25]. The OSPAQ has demonstrated good test-retest reliability for assessing sitting (ICC = 0.89) and standing at work (ICC = 0.90). Validity correlations against ActiGraph accelerometers for occupational sitting and standing (light-intensity activity from the ActiGraph) time were *rho* = 0.65 and *rho* = 0.49, respectively [25]. To calculate minutes per day for both sitting and standing time during work hours, self-reported time spent in each activity was multiplied by the number of self-reported hours worked per day.

The *Workforce Sitting Questionnaire* (WSQ) asks participants to report their time spent sitting 1) while travelling to and from places, 2) while at work, 3) while watching TV, 4) while using a computer at home, and 5) while doing other leisure activities on an average workday and non-workday in the last 7 days [24]. For this study, only time reported sitting while at work in the last 7 days was used, which has previously shown acceptable test-retest reliability (ICC = 0.63) and validity against ActiGraph accelerometers (*rho* = 0.45) for assessing sitting while at work on a workday [24].

Participants also provided information about their gender, age, self-reported height and weight, employment status (full time or part time, number of days worked, and hours worked per week), type of office arrangement (own office or open plan) and highest level of education.

## Analyses

We analyzed the data using SPSS 20.0. The sitting and standing values of the sample at each time point and as measured with the four different methods were described as mean (SD) and as percentage of work time. Outliers exceeding 800 min of occupational sitting time per day were set to missing (OSPAQ: N = 1 at both T0 and T1; WSQ: N = 1 at T0). Unrealistic self-reported work hours (<1.8 h/day) were also set to missing (OSPAQ: N = 3 at T1). Analyses of activPAL and ActiGraph included participants with valid data on at least 2 workdays if working full time or at least 1 workday if working part time. ActivPAL and ActiGraph data were considered valid when the participant wore the device for at least 75 % of their time at work [33].

### Validity

The activPAL was used as the criterion validity measure for occupational sitting and standing time [26]. Spearman’s rho correlations and Bland-Altman plots were calculated for activPAL compared to each of the other three methods. For occupational sitting time, we compared activPAL sitting time at work with ActiGraph sedentary time at work (<100 counts/min), self-reported sitting time at work (WSQ) and self-reported proportion of occupational sitting (OSPAQ). For occupational standing time, we compared activPAL standing time at work with ActiGraph light-intensity activities at work (100–2019 counts/min) and self-reported proportion of standing time (OSPAQ).

The strength of correlation as indicated by Spearman’s *rho* was interpreted as weak (<0.30), low (0.30–0.49), moderate (0.50–0.69), strong (0.70–0.89) or very strong (≥0.90) [34]. To enable comparison of the earlier reported measurement properties of the OSPAQ and WSQ, sitting time from both questionnaires was also compared to sedentary behavior as assessed by the ActiGraph.

### Responsiveness

Responsiveness to change of activPAL, ActiGraph, WSQ and OSPAQ of sitting and standing (except for WSQ) time at work was evaluated using the responsiveness index [35]. The responsiveness index was calculated by dividing absolute intervention group change with the comparison group change SD [21]. The magnitude of the responsiveness index indicates the size of the relative intervention group change compared to the control group variability in the measurement. The responsiveness index was interpreted as small (<0.5), moderate (0.5-0.8) and large (>0.8) change [36].

## Results

Socio-demographic characteristics of the forty-two participants are shown in Table 1. Participants were mostly women (86 %), employed full time (81 %), working in an open-plan office (86 %), with a university education (79 %), and with a body mass index (BMI) within the normal range (50 %). Table 2 presents the mean estimates and percentages of work time for objective and self-report assessments of occupational sitting and standing time at T0, T1 and T2. Average wear time during workhours was 470 minutes for ActiGraph and 458 minutes for activPAL.

**Table 1:** Baseline characteristics of participants in the study

Characteristics	Mean (SD) or N (%)
N	42
Sex (women)	36 (86 %)
Age (years)^a^	38 (11)
Weight (kg)^b^	64 (13)
Height (m)	165 (9)
Body mass index (kg/m^2^)	
Underweight (<18.5)	5 (13 %)
Normal weight (18.5–24.9)	20 (50 %)
Overweight (25.0–29.9)	10 (25 %)
Obese (≥30.0)	5 (13 %)
Working full time	34 (81 %)
Office type	
Own office	6 (14 %)
Open-plan	36 (86 %)
Highest education level	
Completed all years of high school	3 (7 %)
Trade/technical certificate or diploma	6 (14 %)
University	33 (79 %)

^a^Data missing for N = 1 | ^b^data missing for N = 2

**Table 2:** Occupational sitting and standing time at three time points according to four different methods

	6 weeks pre intervention (T0)			2 weeks pre intervention (T1)			3 weeks after introduction sit-stand workstation (T2)		
	n	mean (SD)	%^a^	n	mean (SD)	%^a^	n	mean (SD)	%^a^
Occupational sitting time									
activPAL (min/workday)	35	347 (58)	78	39	360 (74)	78	39	279 (78)	60
OSPAQ (min/workday)	34	391 (88)	80	38	381 (105)	74	41	263 (114)	52
WSQ (min/workday)	33	405 (72)	84	38	386 (94)	76	38	294 (122)	60
ActiGraph ‘sedentary time’ (min/workday)	34	348 (54)	75	39	349 (56)	76	39	366 (46)	76
Occupational standing time									
activPAL (min/workday)	35	45 (27)	10	39	46 (26)	10	39	128 (66)	27
OSPAQ (min/workday)	35	47 (29)	9	39	56 (50)	11	41	160 (76)	33
ActiGraph light-intensity activity (min/workday)	34	98 (28)	21	39	99 (43)	21	39	101 (30)	21

^a^Percent of working time

### Criterion validity for measuring occupational sitting and standing time

Table 3 describes the criterion validity of the ActiGraph, OSPAQ and WSQ, when compared to the activPAL at the three study time points. The ActiGraph showed strong validity for occupational sedentary time at T0 and T1 (*rho* = 0.77 and *rho* = 0.69) and weak validity at T2 (*rho* = 0.19). The OSPAQ had consistently low validity across all three time points for occupational sitting (*rho* T0 = 0.37, *rho* T1 = 0.48 and *rho* T2 = 0.35). The WSQ showed weak validity across all three measurements (*rho* T0 = 0.25, *rho* T1 = 0.29 and *rho* T2 = 0.30).

**Table 3:** Criterion validity for measuring occupational sitting and standing time compared to the activPAL

	6 weeks pre intervention (T0)		2 weeks pre intervention (T1)		3 weeks after introduction sit-stand workstation (T2)	
Outcome	N	Spearman *r*	N	Spearman *r*	N	Spearman *r*
Occupational sitting time^a^						
OSPAQ (min/workday)	31	**0.37**	38	**0.48**	38	**0.35**
WSQ (min/workday)	31	0.25	36	0.29	36	0.30
ActiGraph 'sedentary time' (min/workday)	34	**0.77**	39	**0.69**	38	0.19
Occupational standing time^a^						
OSPAQ (min/workday)	32	0.20	39	0.16	38	**0.68**
ActiGraph light-intensity activity (min/workday)	34	0.25	39	**0.63**	38	0.32

^a^activPAL (min/workday) - reference measurement instrument | bold = significant *p* < 0.05

**Fig. 2:**
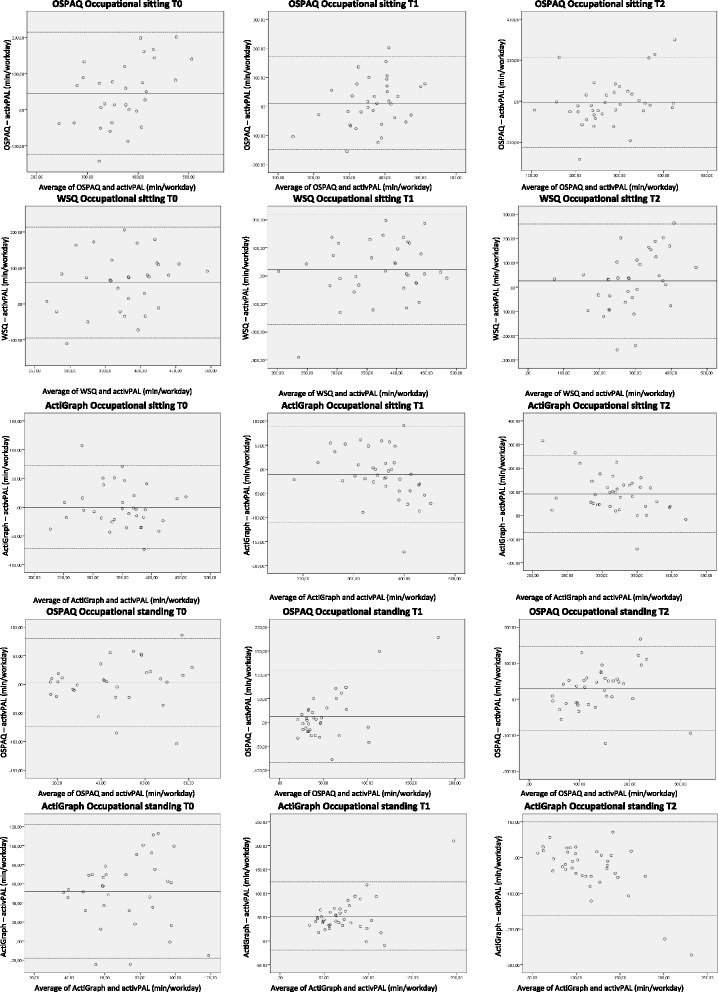
Bland-Altman plots for OSPAQ, WSQ and ActiGraph, when compared to the activPAL for sitting and standing at three study time points. *The solid line represents the mean differences between the two measures and the two dashed lines are the 95 %

For occupational standing time, the OSPAQ showed inconsistent results: weak validity at T0 and T1 (*rho* = 0.20 and 0.16), but moderate and almost strong validity at T2 (*rho* = 0.68). The ActiGraph showed low validity for occupational light-intensity activity time vs activPAL standing time at T0 and T2 (*rho* = 0.25 and 0.32), respectively, but moderate validity at T1 (*rho* = 0.63). The Bland-Altman plots for activPAL compared to all three other measures at the three study time points are presented in Fig. 2.

Spearman correlations for occupational sitting from OSPAQ compared to sedentary time from the ActiGraph monitor were 0.54, 0.47 and 0.23 at T0, T1, T2, respectively. Similarly, the occupational sitting time from the WSQ compared to occupational sedentary time from the ActiGraph showed Spearman correlations of 0.39, 0.50 and 0.30 at T0, T1, T2, respectively (data not shown in tables).

### Responsiveness for measuring change in sitting and standing time at work

Responsiveness to the intervention-induced changes in occupational sitting and standing time is presented in Table 4. The activPAL, OSPAQ, and WSQ showed reductions in occupational sitting time in the intervention group ranging from −114 to −80 min/workday, which was accompanied by similar increases in standing time. The activPAL showed large responsiveness for both occupational sitting (index = 1.1) and standing time (index = 3.7). The ActiGraph showed small responsiveness in both occupational sedentary time and light-intensity physical activity. The OSPAQ showed large responsiveness for both occupational sitting (index = 1.4) and occupational standing time (index = 1.7). The WSQ showed moderate responsiveness (index = 0.7) for occupational sitting time.

**Table 4:** Responsiveness to intervention induced change in occupational sitting and standing time

	Control group (N = 38)			Intervention group (N = 42)			Responsiveness
	Mean change from baseline			Mean change from baseline			index
	N	(T1-T0)	(SD)	N	(T2-T1)	(SD)	
Occupational sitting time							
activPAL (min/workday)	34	7	(73)	38	−80	(107)	1.1
OSPAQ (min/workday)	30	1	(82)	37	−114	(113)	1.4
WSQ (min/workday)	32	−30	(127)	35	−93	(121)	0.7
ActiGraph ‘sedentary time’ (min/workday)	33	2	(54)	38	16	(54)	0.3
Occupational standing time							
activPAL (min/workday)	34	2	(23)	38	83	(65)	3.7
OSPAQ (min/workday)	32	7	(55)	38	96	(91)	1.7
ActiGraph light-intensity activity (min/workday)	33	1	(40)	38	2	(42)	0.1

## Discussion

This study assessed the criterion validity and responsiveness of different methods for measuring occupational sitting and standing time. The ActiGraph showed strong criterion validity against the activPAL when participants were sitting the vast majority of their work time. However, the ActiGraph validity was shown to be weak when participants were standing for a substantial part of their work time. Validity correlations varied from low to moderate for ActiGraph assessed light-intensity activity compared to activPAL-assessed standing time. The OSPAQ and WSQ showed low criterion validity for occupational sitting and standing (OSPAQ only), which is in line with what is commonly found for self-report questionnaires. Both the activPAL and OSPAQ showed large responsiveness to intervention-induced changes in occupational sitting and standing time. Whereas the ActiGraph showed small responsiveness to the intervention-induced changes. Responsiveness was moderate for occupational sitting time for WSQ.

In line with other studies, the activPAL showed high responsiveness to changes in both occupational sitting and standing time [27–29, 37]. A previous study using the same responsiveness method reported an index of only 0.11 for activPAL, but they reported a much smaller reduction in sitting time in their intervention (15 min/d) [21].

The low responsiveness of the ActiGraph, was in line with another study which reported that the ActiGraph did not detect the effects of an intervention to reduce and break up sedentary time in adults [28]. The ActiGraph does not seem to be able to distinguish sitting from standing when worn on the hip and when only the vertical axis with traditional activity cut-points are used. This is not surprising as this traditional data assessment method only uses the vertical axis and neither sitting nor standing are characterized by strong vertical accelerations, which makes it difficult to distinguish between these two behaviors [38]. This is further illustrated by the fact that the ActiGraph assesses sedentary time and light-intensity activity, and not sitting and standing body postures. However, standing is considered to be of light-intensity activity and is mostly misclassified as sedentary time by the ActiGraph. Different placement (i.e. the thigh) and new methods for data reduction and interpretation using raw acceleration data from all three axes are likely to improve the validity and responsiveness of the ActiGraph for assessing occupational sitting and standing time. However, such methods are currently not widely available.

The current study did not only compare the questionnaires to the ActivPAL but also to the ActiGraph, which allowed for comparison to previous ActiGraph based validation studies of these questionnaires. Contrary to the study of Chau et al., the OSPAQ showed lower validity for occupational sitting time (*rho* = 0.23 to 0.54 vs. *rho* = 0.65 in Chau’s study) [25]; the WSQ showed similar low validity (*rho* = 0.30 to 0.50 vs. *rho* = 0.45 in Chau’s study) [24].

The OSPAQ showed strong validity for standing time at T2 and a large responsiveness to change for both sitting and standing. It is not too surprising that standing time correlations at T0 and T1 were low as standing time was small, resulting in overall low scores with low variation between participants, making it difficult for the questionnaire to determine between them, and hence resulting in low correlations. It might also be that, due to the introduction of the sit-stand workstations, participants became more aware of their ‘increased’ standing behavior and, therefore, were better able to estimate their actual time spent standing at work. This possibly makes OSPAQ an inexpensive alternative for an objective assessment method in the evaluation of sit-stand workstation interventions, although it might overestimate effectiveness.

Although the WSQ showed weak validity against the activPAL across all three measurements, the questionnaire showed moderate responsiveness for sitting at work. The WSQ can, therefore, be regarded as useful for ascertaining intervention effectiveness, especially in addition to an objective assessment method by providing domain-specific information.

The main strength of this study was the large intervention effect that allowed testing of the responsiveness of four commonly used assessment methods. Another strength was the ability to not only examine occupational sitting but also standing time, which is increasingly seen as an important alternative to reduce occupational sitting time. A limitation was the selected and relatively small study sample of mainly highly educated women working in a health-related field, which might influence the generalizability of the measurement properties observed here to less well educated populations.

## Conclusions

Future studies aimed at determining differences in occupational sitting and standing time should contemplate using activPAL-type inclinometers as a preferred objective outcome measure. Although, the brief self-report OSPAQ and WSQ questionnaires showed poorer measurement properties than the activPAL, they have shown sufficient validity and responsiveness and would be suitable for use in addition to an objective measure or alone when objective monitoring is not possible. The hip-worn ActiGraph was unable to distinguish between occupational sitting and standing time, when using uniaxial data and traditional cut-points for sedentary time and light-intensity activity. Further validation and exploration of responsiveness of both objective and self-reported occupational sitting and standing time in larger and more heterogeneous samples and with different intervention strategies would help to determine the relevance of these findings in other settings and populations.

## Abbreviations

BMI: body mass index
IPAQ: International Physical Activity Questionnaire
OSPAQ: Occupational Sitting and Physical Activity Questionnaire
PAST: Past-day Adults’ Sedentary Time
WSQ: Workforce Sitting Questionnaire
